# Pregnenolone Rescues Schizophrenia-Like Behavior in Dopamine Transporter Knockout Mice

**DOI:** 10.1371/journal.pone.0051455

**Published:** 2012-12-11

**Authors:** Peiyan Wong, Cecilia Chin Roei Chang, Christine E. Marx, Marc G. Caron, William C. Wetsel, Xiaodong Zhang

**Affiliations:** 1 Neuroscience and Behavioral Disorders Program, Duke-NUS Graduate Medical School Singapore, Singapore, Singapore; 2 Durham VA Medical Center, Department of Veterans Affairs, Durham, North Carolina, United States of America; 3 Department of Psychiatry and Behavioral Sciences, Duke University Medical Center, Durham, North Carolina, United States of America; 4 Department of Cell Biology, Duke University Medical Center, Durham, North Carolina, United States of America; 5 Department of Neurobiology, Duke University Medical Center, Durham, North Carolina, United States of America; 6 Department of Physiology, National University of Singapore, Singapore, Singapore; Chiba University Center for Forensic Mental Health, Japan

## Abstract

Pregnenolone belongs to a class of endogenous neurosteroids in the central nervous system (CNS), which has been suggested to enhance cognitive functions through GABA_A_ receptor signaling by its metabolites. It has been shown that the level of pregnenolone is altered in certain brain areas of schizophrenic patients, and clozapine enhances pregnenolone in the CNS in rats, suggesting that pregnenolone could be used to treat certain symptoms of schizophrenia. In addition, early phase proof-of-concept clinical trials have indicated that pregnenolone is effective in reducing the negative symptoms and cognitive deficits of schizophrenia patients. Here, we evaluate the actions of pregnenolone on a mouse model for schizophrenia, the dopamine transporter knockout mouse (DAT KO). DAT KO mice mirror certain symptoms evident in patients with schizophrenia, such as the psychomotor agitation, stereotypy, deficits of prepulse inhibition and cognitive impairments. Following acute treatment, pregnenolone was found to reduce the hyperlocomotion, stereotypic bouts and pre-pulse inhibition (PPI) deficits in DAT KO mice in a dose-dependent manner. At 60 mg/kg of pregnenolone, there were no significant differences in locomotor activities and stereotypy between wild-type and DAT KO mice. Similarly, acute treatment of 60 mg/kg of pregnenolone fully rescued PPI deficits of DAT KO mice. Following chronic treatment with pregnenolone at 60 mg/kg, the cognitive deficits of DAT KO mice were rescued in the paradigms of novel object recognition test and social transmission of food preference test. Pregnenolone thus holds promise as a therapeutic candidate in schizophrenia.

## Introduction

Schizophrenia is a neuropsychiatric disorder that affects approximately 1% of the world’s population and is characterized by a clinical manifestation of psychotic symptoms, such as auditory hallucinations and delusions. However, the largest contributing factors to the incapacitating nature of the illness are the negative symptoms and cognitive impairments, due to the strong correlation with decreased functionality and quality of life [1], [2], [3], [4], [5], [6]. The development of antipsychotic drugs, the exacerbating effect of NMDA antagonists on schizophrenia patients, as well as genetic and animal studies strongly suggest that dysregulation in neurotransmitter homeostasis, such as dopamine, glutamate and GABA, is implicated in the pathophysiology of schizophrenia [7], [8], [9]. First- and second- generation antipsychotics are generally effective in treating the positive symptoms of schizophrenia. However, the paucity of effective interventions for cognitive symptoms in patients with schizophrenia emphasizes the necessity to develop other therapeutic agents that are efficacious against these symptoms.

Neurosteroids are synthesized in the central nervous system (CNS), and accumulate in the brain at physiologically relevant concentrations [10], [11]. In rodents, these neurosteroids are present in the CNS in higher levels than in the periphery, and are known to have diverse actions in the CNS, including effects on cognition, anxiety and depression [12], [13], [14], [15], [16], [17].

Previous studies have shown that pregnenolone levels are altered in the parietal and cingulate cortices of postmortem brain tissues from schizophrenia patients [18], suggesting that pregnenolone may be involved in the psychoneurological basis of the disorder. Clinical trials using pregnenolone as a therapeutic agent for schizophrenia have been encouraging to date, showing increases in attention, verbal and working memory, and decreases in negative symptoms [19], [20]. Pregnenolone is also generally well-tolerated by patients [19], [20], [21], with significantly decreased positive symptoms and extrapyramidal side-effects [21].

Hyperdopaminergic function has been implicated in many psychiatric disorders including schizophrenia. Because dopaminergic homeostasis is maintained by dopamine transporter to uptake released dopamine from synaptic cleft, dopamine transporter knockout mice (DAT KO) exhibit increased dopaminergic tone, leading to hyperactive and stereotypic behaviors [22]. DAT KO mice also show impaired sensorimotor gating [23], [24], spatial learning and working memory [25], [26], [27], all of which mirror certain symptoms of schizophrenia [28]. In addition, dopamine receptor functions are altered in DAT KO mice, as indicated by reduced dopamine D2 autoreceptor function [29] and attenuation of hyperlocomotor activities upon treatment with dopamine D1, D2 antagonists [30]. The alleviation of sensorimotor gating deficit in DAT KO mice by D2 antagonist [23] and atypical antipsychotics [24] further suggests the dysfunction of dopamine receptor signaling in schizophrenia. Moreover, dysfunction of AKT/GSK3 pathway has been implicated in schizophrenia [31]. In DAT KO mice, GSK3 activities were elevated through dopamine D2 receptor signaling [30]. Therefore, DAT KO mice have been considered a very useful animal model to study certain aspects of schizophrenia [28]. In the current study, we used DAT KO mice to assess the potential of pregnenolone as a therapeutic agent of schizophrenia.

## Materials and Methods

### Animals

Adult (8–10 weeks of age) male and female WT and DAT KO mice were generated by breeding heterozygous DAT mice that were on a congenic C57BL/6J background. Mice were genotyped by PCR using primers DA4-3B/R (5′- TGT CTC CAC CTT CCT AGC ACT AAC TAG C-3′), DAT-Neo-B (5′- ACC CGT GAT ATT GCT GAA GAG CTT G-3′) and DA5B/F (5′- TCA TCT TGG TCA AGG AGC AGA ATG GAG-3′) in buffer containing 100 mM Tris (pH 8.5), 5 mM EDTA, 0.2% sodium dodecyl sulfate (SDS), and 200 mM NaCl under conditions as previously described [22]. Mice were housed in a pathogen-free environment, maintained under 22°C, 55% humidity, with food and water provided *ad libitum*, on a 12-hr light/dark cycle (lights on at 0700 h). All experiments were conducted in accordance with national guidelines for the care and use of laboratory animals for scientific purposes with approved protocols from the Institutional Animal Care and Use Committees of Duke University and Duke-NUS Graduate Medical School Singapore.

### Drug Preparation

Haloperidol (HAL) (Sigma-Aldrich, St. Louis, MO) and clozapine (CLZ) (Tocris Biosciences, UK) were dissolved in a minimal amount of 0.1 M HCl and glacial acetic acid, respectively, before diluting with distilled water. Pregnenolone (Preg) (Sigma-Aldrich) was dissolved in a minimal volume of 0.5% SDS and resuspended in peanut oil. The vehicle solution was a minimal amount of 0.5% SDS in peanut oil. All injections were performed with a 5 ml/kg injection volume.

### Activity in the Open Field

Locomotor, rearing, and stereotypical activities were monitored for individual mice using an automated Omnitech Digiscan apparatus (21×21×30 cm; AccuScan Instruments, Columbus, OH) under ∼180 lux illumination. Mice were placed into the apparatus for 30 min to obtain baseline activity, then injected (i.p.) with vehicle, 0.2 mg/kg HAL, 2.0 mg/kg CLZ, or 30 or 60 mg/kg Preg, and immediately returned to the open field for 120 min. Locomotion was measured as total distance traveled, rearing as vertical activity, and stereotypical activity as the numbers of consecutive beam-breaks (<1 sec).

### Prepulse Inhibition Test

Prepulse inhibition (PPI) of the acoustic startle response was conducted as described [23], [32] using SR-LAB startle chambers (San Diego Instruments, San Diego, CA). Mice were administered (i.p.) vehicle, or 30 or 60 mg/kg Preg and were placed into the plexiglas cylinder and apparatus for 10 mins of habituation. Startle trials consisted of a 40 ms burst of 120dB white-noise; pre-pulse trials consisted of a 20 ms pre-pulse stimulus that was 4, 8, or 12dB above the white-noise background (64dB), followed 100 ms later by the 120dB startle stimulus. Non-stimulus or null trials consisted of the 64dB white-noise background. PPI responses were calculated as a percentage score for each intensity of pre-pulse, where %PPI = [1–(pre-pulse trials/startle-only trials)]*100.

### Novel Object Recognition Test

Mice were administered (s.c) vehicle or 60 mg/kg Preg once daily for 14 consecutive days. Twenty-four hrs later, mice were exposed to two identical objects for 10 min as described (39). A test for short term memory (STM) was conducted 20 min after training. Assessments of long-term memory (LTM) and remote memory were conducted 24 hrs and 14 days after training, respectively. The objects were chosen based on similarities in dimensions and complexity. Tests were conducted in an acrylic box (20.32 cm×40.5 cm×16 cm) that was cleaned, together with the objects with 70% ethanol after each subject was tested. Time spent with an object was defined as the duration and number of contacts was defined as the bouts when the mouse is oriented towards the object and within half a body length of that object, and could include sniffing, touching, or climbing on the object. These tests were video-recorded and scored using JWatcher (UCLA, Los Angeles, CA), by an observer blind to the genotypes and treatment of the animal. Preference scores were calculated as (Time spent with novel object – Time spent with familiar object)/(Total time spent with both objects). Positive scores indicated preferences for the novel object, negative scores showed preferences for the familiar object, and scores approaching zero denoted no preference for either object.

### Social Transmission of Food Preference Test

Mice were injected (s.c.) with vehicle or 60 mg/kg Preg daily for 14 consecutive days. Animals were food deprived immediately after the last injection. Social transmission of food preference tests were conducted as described [33], [34]. The familiar diet was composed of 1% ground oregano (McCormick & Co. Inc, Hunt Valley, USA) in standard mouse chow (5LJ5 Lab Diet Formula; PMI Nutrition International, USA). Novel diets for STM, LTM, and remote memory tests were flavoured with 1% ground thyme, marjoram, or cumin (McCormick & Co. Inc, USA), respectively. Briefly, mice were placed on food restriction for 16–18 hrs on the day prior to training. A demonstrator mouse was allowed to consume a flavoured diet for 30 min and then was returned to its home cage to interact with the tester mice for 20 min. Tester mice were then exposed to the familiar and a novel diet as a test of STM, and the consumption of each diet was monitored. Animals were tested subsequently at 24 hr and 14 days, respectively, to assess LTM and remote memory. Preference scores were calculated as (Amount of familiar diet consumed – Amount of novel diet consumed)/(Total amounts of both diets consumed). Positive preference scores indicated preference for the familiar demonstrator diet, negative scores denoted preferences for the novel diet, and scores approximating zero signified no preference.

### Statistical Analyses

Data were analyzed using SPSS (SPSS, Chicago, IL). The data are presented as means ±SEM. Open field locomotor activities across time, prepulse inhibition, novel object recognition and social transmission of food preference tests were analysed using a mixed factorial design ANOVA. Between subjects factors for all tests were genotype and treatment. Within subjects factors for the open field activities were time, for PPI was inhibition across prepulse intensities, and for object recognition memory and social transmission of food preference data were test days. Cumulative activities in the open field, and null and startle activity in PPI were analyzed by two-way ANOVA, with the between subjects factors being genotype and treatment. Preference scores in the novel object recognition and social transmission of food preference tests were analyzed versus “0” value using a one-sample T test. Bonferroni corrected pair-wise comparisons were used as the *post-hoc* tests. A *p*<0.05 was considered significant.

## Results

### Pregnenolone Reduces Hyperactivity in DAT KO Mice

**Figure 1:**
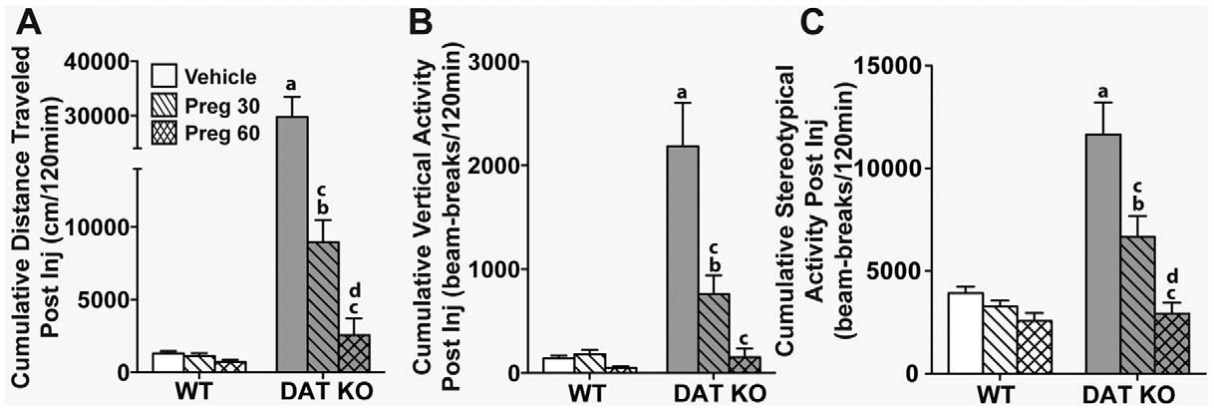
Dose-dependent effects of pregnenolone, haloperidol and clozapine on activities of WT and DAT KO mice in the open field. (A–C) Cumulative distance traveled (A), cumulative vertical activity (B), and cumulative stereotypical activities (C) were monitored over a 2 h period following injection of vehicle, or 30 or 60 mg/kg Preg. N = 10–15 mice/genotype/treatment condition; ^a^ *p*<0.05, WT-Veh versus KO-Veh; ^b^ *p*<0.05, WT-Preg30 versus KO-Preg30; ^c^ *p*<0.05, within groups versus Veh; ^d^ *p*<0.05, within groups Preg30 versus Preg60.

To better visualize the effects, locomotor, rearing, and stereotypical activities were aggregated separately over the 30 min baseline period (0–30 min) and the 2 h period (31–150 min) after drug administration. When cumulative baseline activity was analyzed, a two-way ANOVA across genotype and treatment revealed significant main effects of genotype for locomotion (*F*_(1,53)_ = 156, *p<*0.0001), rearing (*F*_(1,53)_ = 31.4, *p<*0.0001), and stereotypical activity (*F*_(1,53)_ = 14.1, *p<*0.0001), but no significant main effects for treatment and no significant interactions were obtained. As expected (33), the cumulative baseline activities were significantly higher in DAT KO than WT mice (data not shown). For the cumulative post-injection period, a two-way ANOVA across genotype and treatment observed significant main effects of genotype (locomotion: *F*_(1,53)_ = 80.3, *p*<0.0001; rearing: *F*_(1,53)_ = 33.1, *p*<0.0001; and stereotypy: *F*_(1,53)_ = 31.0, *p*<0.0001) and treatment (locomotion: *F*_(2,53)_ = 34.1, *p*<0.0001; rearing: *F*_(2,53)_ = 15.3, *p*<0.0001; and stereotypy: *F*_(2,53)_ = 17.9, *p*<0.0001), and a significant genotype by treatment interaction (locomotion: *F*_(2,53)_ = 31.7, *p*<0.0001; rearing: *F*_(2,53)_ = 13.1, *p*<0.0001; and stereotypy: *F*_(2,53)_ = 9.61, *p*<0.0001). For locomotion, rearing, and stereotypical activities, Bonferroni corrected pair-wise comparisons showed that activities in WT mice were unaffected by 30 or 60 mg/kg Preg (Fig. 1A–C). By contrast, DAT KO mice treated with 30 mg/kg Preg significantly decreased their activities compared to those treated with vehicle (*p*<0.0001). The 60 mg/kg dose further reduced their locomotor (*p*<0.05) and stereotypical (*p*<0.01) hyperactivities compared to the 30 mg/kg dose. Additionally, the 60 mg/kg dose attenuated all three activities of DAT KO mice relative to the vehicle control (*p*s<0.0001) and to levels that were comparable to the WT vehicle control. Together, these data indicate that Preg reduced the hyperactivities of the DAT-KO mice in a dose-dependent fashion, while exerting little effect on WT activities.

### Responses to Pregnenolone, Haloperidol, and Clozapine are Similar

**Figure 2:**
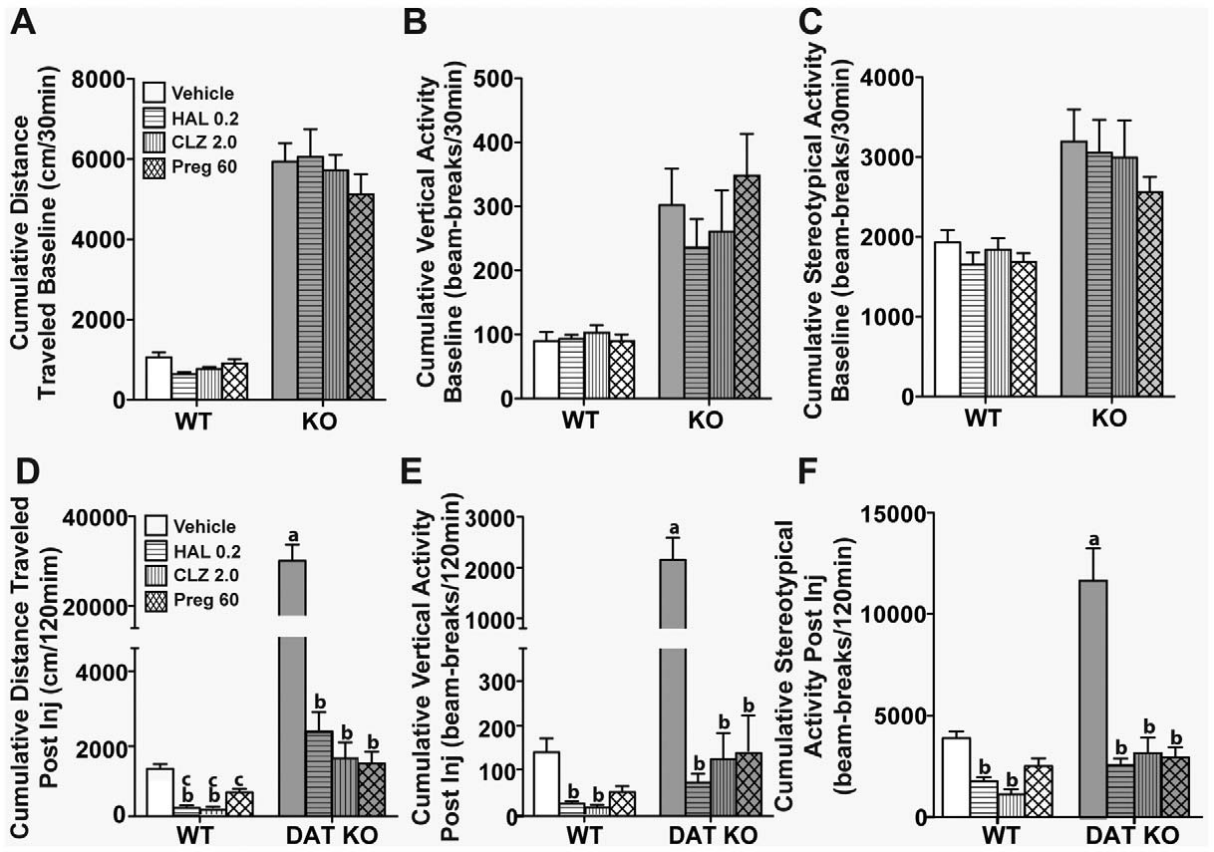
Effects of pregnenolone, haloperidol and clozapine on activities of WT and DAT KO mice in the open field. Baseline activities were monitored over 30 min, the mice were injected (i.p.) with vehicle, or 30 or 60 mg/kg pregnenolone (Preg) and returned immediately to the open field for 2 h. Cumulative distance traveled (A), vertical activity (B), and stereotypical activities (B) are shown. (D–F) Cumulative post-injection activities after WT and DAT KO mice were administered (i.p.) vehicle, 0.2 mg/kg haloperidol (HAL), 2.0 mg/kg clozapine (CLZ), or 60 mg/kg Preg, and monitored for locomotor (D), rearing (E), and stereotypical (F) activities. N = 9–15 mice/genotype/treatment condition, ^a^ *p*<0.05, WT-Veh versus KO-Veh; ^b^ *p*<0.05, within groups versus Veh; ^c^ *p*<0.05, within groups HAL or CLZ versus Preg60.

Haloperidol (HAL) and clozapine (CLZ), at the doses of 0.2 mg/kg and 2.0 mg/kg respectively, were used as positive controls to compare the effects of Preg on DAT KO hyperactivity. When baseline activities were collapsed over time (30 min), a two-way ANOVA across genotype and treatment revealed significant main effects for genotype for locomotor (*F*_(1,69)_  = 238, *p<*0.0001), rearing (*F*_(1,69)_ = 31.7, *p<*0.0001), and stereotypical activities (*F*_(1,69)_ = 27.2, *p<*0.0001), whereas the genotype by treatment interaction was not significant. Within genotype, baseline activities were similar among the treatment groups and, as expected (33), were significantly higher in DAT KO than in WT mice (*p*s<0.05) (Fig. 2A–C). Following the drug injection, a two-way ANOVA across genotype and treatment observed significant main effects of genotype (locomotion: *F*_(1,69)_ = 61.6, *p*<0.0001; rearing: *F*_(1,69)_ = 27.9, *p*<0.0001; and stereotypy: *F*_(1,69)_ = 29.3, *p*<0.0001) and treatment (locomotion: *F*_(3,69)_ = 32.5, *p*<0.0001; rearing: *F*_(3,69)_ = 17.6, *p*<0.0001; and stereotypy: *F*_(3,69)_ = 22.9, *p*<0.0001), and a significant genotype by treatment interaction (locomotion: *F*_(3,69)_ = 28.6, *p*<0.0001; rearing: *F*_(3,69)_ = 14.1, *p*<0.0001; and stereotypy: *F*_(3,69)_ = 8.81, *p*<0.0001). For locomotion (Fig. 2D), both HAL and CLZ significantly decreased locomotor activities of WT (*p*<0.0001) and DAT KO (*p*<0.0001) mice relative to their respective vehicle controls. Although these antipsychotic drugs were more effective in suppressing locomotion in WT mice than 60 mg/kg Preg (*p*s<0.01), no differences were observed among the drugs and Preg in the DAT KO mice. With respect to rearing (Fig. 2E), activities in WT and DAT KO mice were significantly decreased by HAL or CLZ treatments (*p*s<0.0001) relative to their respective controls, and the rearing activity in the DAT KO mice were reduced to levels similar to those of the WT vehicle-controls. As noted above, 60 mg/kg Preg exerted no effects on WT rearing, whereas rearing in DAT KO mice was suppressed (*p*<0.0001). For stereotypical activity (Fig. 2F), both HAL and CLZ reduced activity in WT mice (*ps*<0.0001), whereas the Preg effect was not significant. By comparison, all three drugs depressed the stereotypies in DAT KO mice (*ps*<0.0001) to the levels of the WT vehicle controls. Collectively, these findings show that 60 mg/kg Preg is as efficacious in suppressing the hyperactivity in DAT KO mice as 0.2 mg/kg HAL and 2 mg/kg CLZ. However, Preg did not affect the activities of WT mice, unlike the antipsychotic drugs.

### Pregnenolone Rescues Prepulse Inhibition in DAT KO Mice

**Figure 3:**
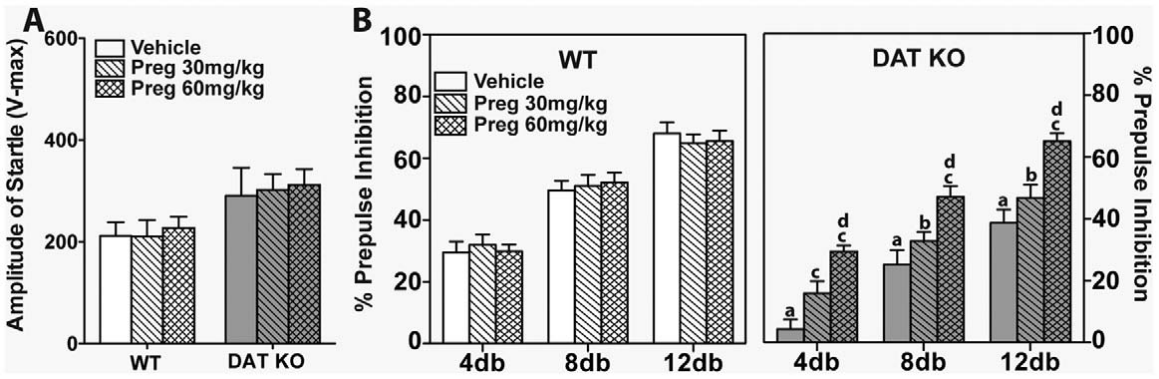
Pregnenolone rescues PPI in DAT KO mice. WT and DAT KO mice were injected (i.p.) with vehicle, or 30, or 60 mg/kg Preg and were tested in PPI 5 min later. (A) Amplitude of the startle responses of WT and DAT KO mice. (B) PPI levels of WT and DAT KO mice. White bars represent WT and grey bars represent DAT KO performance. N = 9–14 mice/genotype/treatment condition; ^a^ *p*<0.05, WT-Veh versus KO-Veh; ^b^ *p*<0.05, WT-Preg30 versus KO-Preg30; ^c^ *p*<0.05, within groups versus Veh; ^d^ *p*<0.05, within groups Preg30 versus Preg60.

To determine whether Preg could rescue the PPI deficiency in DAT KO mice, animals were given 30 or 60 mg/kg Preg. No genotype differences were detected in percentage of null activity (WT: 3.2–9.1%; DAT KO: 3.0–9.5%). A two-way ANOVA for startle responses found significant main effects of genotype (*F*_(1,58)_ = 7.57, *p*<0.01), due to increased responses of the DAT KO mice; however, the treatment effect and genotype by treatment interaction were not significant (Fig. 3A). A mixed-design ANOVA for prepulse inhibition across genotype, treatment and prepulse intensities revealed significant between subjects main effects of genotype (*F*_(1,63)_ = 47.8, *p*<0.0001) and treatment (*F*_(2,63)_ = 11.6 *p*<0.0001), with a significant genotype by treatment interaction (*F*_(2,63)_ = 11.9, *p*<0.0001). There was also a significant within subjects main effect of prepulse intensities (*F*_(2,126)_ = 269, *p*<0.0001), although all interaction terms for prepulse intensity, genotype and treatment were not significant. Bonferroni corrected pair-wise comparisons revealed that prepulse-dependency of PPI was observed under both genotype and treatment conditions: 4 versus 8dB (*p*s<0.001), 8 versus 12dB (*p*s<0.001), and 4 versus 12dB (*p*s<0.001) (Fig. 3B). Thirty or 60 mg/kg Preg exerted no effects on PPI in WT mice compared to vehicle controls (Fig. 2, left). Compared to WT controls, PPI was decreased at all prepulse intensities in vehicle-treated DAT KO mice (*p*s<0.0001) (Fig. 2, right). Although 30 mg/kg Preg increased the 4dB response in DAT KO mice, responses to the 8 and 12dB prepulses were still deficient compared to WT controls (*p*s<0.05). In DAT KO mice, the 60 mg/kg dose fully rescued PPI at all three prepulse intensities to the levels of the WT mice, and this increase was, at all intensities, significantly above that of the vehicle- (*ps*<0.001) or 30 mg/kg Preg- treated DAT KO mice (*p*s<0.05). Taken together, these findings show that 60 mg/kg Preg can normalize PPI in DAT KO mice.

### Pregnenolone Normalizes Deficiencies in Episodic Memory

**Figure 4:**
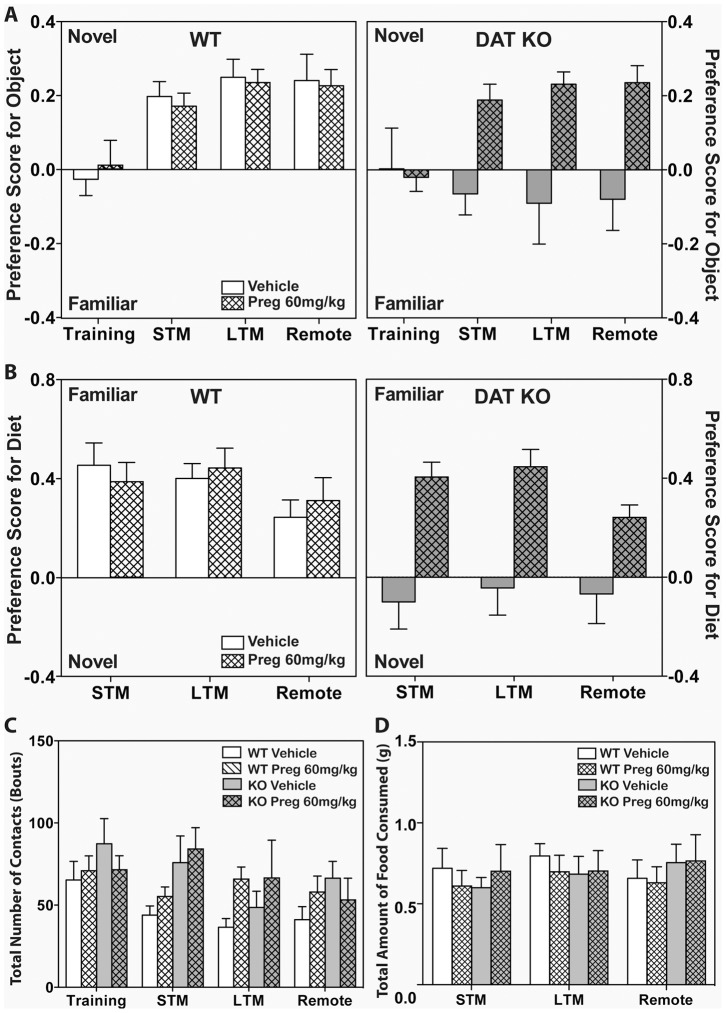
Pregnenolone normalizes the episodic memory deficits in DAT KO mice. WT and DAT KO mice were injected (s.c.) with vehicle or 60 mg/kg Preg for 14 consecutive days and were tested in the novel object recognition (A) and social transmission of food preference (B) tests for short-term (STM), long-term (LTM), and remote memory. Number of contacts with the novel and familiar objects in the novel object recognition test (C) and the amount of food consumed in the social transmission of food preference test (D) were analysed. For the novel object recognition test, N = 9–12, and for the social transmission of food preference test, N = 9–11.

The effects of 14 days of administration (s.c.) of 60 mg/kg Preg were examined in DAT mice in the novel object recognition test. A mixed-design ANOVA for preference for the novel object across genotype, treatment and test day revealed significant between subjects main effects of genotype (*F*_(1,31)_ = 8.18, *p*<0.01) and treatment (*F*_(1,31)_ = 8.05, *p*<0.01), with a significant genotype by treatment interaction (*F*_(1,31)_ = 10.6, *p*<0.01). There was significant within subjects effect of test day (*F*_(3,93)_ = 6.28, *p*<0.01), but no interactions between test day, genotype and treatment. During training, neither genotype nor treatment group showed any preference for either identical object (Fig. 4A). During testing, vehicle-treated WT mice preferred the novel object to equal extents across each of the 3 test days and this preference did not change with Preg treatment. By comparison, the vehicle-treated DAT KO mice displayed no preference for either object during each of the 3 test days, with no significant changes across these days. Hence, preference scores for DAT KO vehicle-controls were significantly lower than those of the vehicle- and Preg-treated WT mice (*p*s<0.05) for all the test days. Compared to their vehicle controls, the Preg-treated DAT KO mice demonstrated preferences for the novel objects across each of the test days (*ps*<0.01) and these scores were not statistically different from those of the vehicle- or Preg-treated WT mice on each of the 3 test days. A one-sample T test showed that the preference scores of the vehicle- and Preg-treated WT groups, and Preg-treated KO group for STM, LTM and remote test days were significantly different from 0 (t(>9)<2.93, p<0.05).

To ensure that the deficiencies in the DAT KO vehicle-controls could not be attributed to reduced object exploration, the number of object contacts was calculated (Fig. 4C). There were only significant within subjects effect of test-day (*F*_(3,111)_ = 3.61, *p*<0.05), with no other significant main effects or interactions. However, the Bonferroni comparisons failed to detect any differences across test days. These findings show that the reduction is novel object preference by the DAT KO mice cannot be attributed to genotype differences in object interactions. Collectively, these results suggest that 14 days of treatment with 60 mg/kg Preg can normalize the deficits of the DAT KO mice in the novel object recognition test.

### Pregnenolone Normalizes the Deficiency in the Social Transmission of Food Preference

In the social transmission of food preference test, WT and DAT KO mice were given 60 mg/kg Preg for 14 days prior to testing. Mice were tested for STM, LTM, and remote social memory after interaction with the demonstrator mouse. A mixed-design ANOVA for preference for the familiar diet across genotype, treatment and test day showed significant between subjects main effects of genotype (*F*_(1,18)_ = 13.3, *p*<0.01) and treatment (*F*_(1,18)_ = 10.2, *p*<0.01), with a significant genotype by treatment interaction (*F*_(1,18)_ = 6.64; *p*<0.05). The within subjects analysis failed to find any significant effects of test day or interactions with test day (Fig. 4B). Bonferroni comparisons noted that both vehicle- and Preg-treated WT mice preferred the familiar diet on each of the 3 test days and this preference did not change between the 2 treatment conditions. By contrast, vehicle-treated DAT KO mice demonstrated no preference for either diet on any of the test days and their preference scores were significantly lower than those for the vehicle- and Preg-treated WT animals (*p*s<0.05). Interestingly, compared to the vehicle-controls the Preg-treated DAT KO mice preferred the familiar diet on all 3 test days (*ps*<0.05) and their preference scores were similar to those of the vehicle- and Preg-treated WT animals. A one-sample T test showed that the preference scores of the vehicle- and Preg-treated WT groups, and Preg-treated KO group for the 3 test days were significantly different from 0 (t(>9)<2.83, p<0.05), except for vehicle- and Preg-treated WT groups during LTM, where the scores did not significantly differ from 0 (t(>9)<1.99, p<0.1).

To ensure that the deficiencies in the DAT KO vehicle-controls were not due to motivational differences between genotypes, the amount of food consumed during each of the 3 test days was analyzed (Fig. 4D). A RMANOVA revealed no significant main effects and no significant interactions. Hence, the motivation for consuming the different diets appears to be similar between the genotypes. Together, these findings indicate that 14-days of treatment with 60 mg/kg Preg can normalize the deficits of the DAT KO mice in the social transmission of food preference test.

## Discussion

The effectiveness of pregnenolone, a prohormone that is synthesized directly from cholesterol in the brain, in alleviating the schizophrenia-like symptoms of DAT KO mice were characterized in this study. DAT KO mice exhibit various phenotypes that recapitulate certain symptoms of schizophrenia [22], [23], [24], [26], [27]. DAT KO mice consistently showed increased locomotion, rearing activity and stereotypic activity in the open field compared to WT mice [22], [35], [36]. Acute administration of pregnenolone suppressed the open field activities of DAT KO mice in a dose-dependent manner. At 60 mg/kg of pregnenolone, there was no significant difference in the open field activities between WT and DAT KO mice (Fig. 1). Unlike haloperidol and clozapine, 60 mg/kg pregnenolone did not adversely affect WT mice (Fig. 1 and 2). In addition, the PPI deficiency of DAT KO mice was rescued by acute administration of 60 mg/kg pregnenolone (Fig. 3). When chronically administered, pregnenolone at 60 mg/kg was effective in alleviating the cognitive deficits of DAT KO mice in both paradigms of novel object recognition and social transmission of food preference tests (Fig. 4). These results indicate that increasing the circulating levels of pregnenolone is able to effectively alleviate both positive and negative schizophrenia-like symptoms in DAT KO mice, without adversely affecting WT controls.

Neurosteroids, including pregnenolone and its downstream products (e.g. allopregnanolone, dehydroepiandrosterone (DHEA) and pregnenolone sulphate) [37], [38], [39], [40], have been shown to improve learning and memory [41] through their respective actions on NMDA and GABA_A_ receptors [18], [19], [20], [42], [43], [44]. In addition, neurosteroids, such as pregnenolone, DHEA and DHEA sulphate, are agonists for the endoplasmic reticulum sigma-1 receptor, which has been implicated in the pathophysiology of psychiatric disorders. [45], [46]. Therefore, it seems that sigma-1 receptor may play a role in the mechanisms of antipsychotic-like action of pregnenolone. To our knowledge, this is the first study that characterized the effects of acute, systemic pregnenolone administration in DAT KO mice, which is an established mouse model for schizophrenia [28]. In a previous study, progesterone, a downstream metabolite of pregnenolone, was shown to suppress the hyperactivity of DAT KO mice [47]. Another metabolite of pregnenolone, allopregnanolone, which is known to have GABA_A_ modulatory properties, has also been shown to increase inhibition of prepulse startle in rats [48]. In comparison to the first- and second- generation antipsychotics, such as haloperidol and clozapine, pregnenolone is as effective in suppressing the hyperactivity and rescuing the PPI deficits of DAT KO mice [23], [24], [35], which suggests that pregnenolone may exert similar effects on dopamine signaling pathways. These findings, coupled with the current results, suggest that acutely administered pregnenolone may modulate dopamine signaling, possibly through direct regulatory mechanisms at the NMDA or GABA_A_ receptors, or indirectly through downstream metabolites, such as allopregnanolone, to normalize the behaviors of DAT KO mice in the open field and PPI.

Cognitive deficits are one of the major debilitating effects in schizophrenia. Previous studies have shown that pregnenolone is effective in improving memory in rodent models [41], [49], [50], [51]. For example, pregnenolone improves the performance of mice in the T-maze [41], [52]. In addition, pregnenolone sulfate, a downstream metabolite of pregnenolone, has neuroprotective effects against learning and memory deficits, possibly through NMDA receptor modulation [38], [53], [54], [55], [56], [57]. Another neuroactive metabolite of pregnenolone, DHEA, also has memory enhancing and anti-amnesic effects [58], [59]. It has previously been shown that DAT KO mice exhibit cognitive deficits in terms of impaired memory and discriminative abilities [35], [60]. Congruent to previous studies, our results showed that DAT KO mice displayed opposite preferences to WT mice in the novel object recognition and social transmission of food preference tests. Following chronic treatment with 60 mg/kg pregnenolone, DAT KO mice showed similar preferences to WT mice for the novel object and familiar diet, indicating a rescue in memory function and discriminative abilities. The results from the current study, coupled with previous findings, suggest that pregnenolone, when administered long-term, can restore the impaired memory function of DAT KO mice to the levels of their WT counterparts, either through direct regulation or through one of its many neuroactive downstream products.

Dysregulation of brain pregnenolone level has been implicated in a number of brain diseases, such as schizophrenia [18], [61], Alzheimer’s disease [10], [62], [63], bipolar disorder [18], [64] and depression [65], [66], suggesting that changes in neurosteroid levels may play a role in the neurobiology of these disorders. Furthermore, an atypical antipsychotic, clozapine, which is often used in patients whom do not respond to other medications, has been shown to increase pregnenolone levels in the rat hippocampus [11]. Another antipsychotic drug, olanzapine, has been shown to increase allopregnanolone in rodent cerebral cortex [67]. These data suggest that neurosteroid induction may contribute to the clinical actions of atypical antipsychotics.

Administration of pregnenolone to human patients in early phase, proof-of-concept clinical trials have been shown to improve negative and cognitive symptoms [19], [21]. Moreover, pregnenolone was well-tolerated by patients, and may be suitable for use as a therapeutic agent [19]. However, there is a gap between clinical studies and animal studies, because most of the results of the animal studies are obtained from i.c.v. administration of pregnenolone [41], [68]. In this study, pregnenolone was administered to the DAT KO mice through a more systemic route, i.p. or s.c., in an attempt to mirror the consumption route in human patients somewhat more closely. In addition, most of the previous animal studies involved WT rodent models or a pharmacologically induced disease state model [52]. In contrast, this study utilizes a rodent model that potentially reflects positive, negative and cognitive symptoms of schizophrenia. As such, this study may thus have multifaceted relevance to the pathophysiology of schizophrenia.

Finally, our study has shown for the first time that pregnenolone, when administered acutely, is effective in calming some positive schizophrenia-like symptoms in the DAT KO mice, such as the psychomotor agitation and stereotypy, and rescuing PPI deficits. Furthermore, when administered chronically, pregnenolone ameliorates the cognitive deficits of DAT KO mice. It is noteworthy that the long-lasting improvement of cognitive functions in this study (Fig. 3) may be contributed by the duration of chronic treatment, which is longer than any pilot studies in human so far [19].

Although our current results are promising, a few questions remain to be addressed for future systematic studies on the effects of pregnenolone. First, conversion of pregnenolone to its neuroactive metabolites, such as allopregnanolone, DHEA and pregnenolone sulphate, requires a number of enzymes, such as P450c17 (17α-hydrolase), 3β-hydroxysteroid dehydrogenase (3β-HSD), 5α-reductase, and pregnenolone sulfotransferase [18]. Therefore, the respective neuronal activities of these enzymes in DAT KO mice as compared to WT mice are of particular interest. Second, pregnenolone was delivered by i.p. or s.c. injection in this study to resemble common delivery routes, the exact concentrations of pregnenolone and its neuroactive metabolites in different brain regions need to be analyzed to understand the mechanism of action. Third, it is unclear which receptor signaling pathway(s) is involved in the effect of pregnenolone in this study. DAT KO mice exhibit hyperdopaminergic function, whereas glutamatergic function seems to be intact [69]. Further studies using specific inhibitors, such as 5α-reductase inhibitor to block the conversion to allopregnanolone [70], or treat DAT KO mice with pregnenolone sulphate, DHEA, allopregnanolone, respectively may provide insights into the action of pregnenolone. While future studies are required to elucidate the mechanisms by which pregnenolone acts upon to alleviate schizophrenia-like symptoms, the results of the current study suggest that pregnenolone may be a suitable and promising therapeutic agent for certain symptoms of schizophrenia.
